# From an election to an insurrection: Investigating differential engagement and sentiment in the #defundthepolice and #defendthepolice network on Twitter

**DOI:** 10.1371/journal.pone.0289041

**Published:** 2024-03-21

**Authors:** Bianca Wirth, Megan Evans

**Affiliations:** Department of Sociology and Criminology, Pennsylvania State University, University Park, PA, United States of America; University of Vermont, UNITED STATES

## Abstract

Social movements and their respective countermovements have evolved to use online social media platforms to recruit followers, share pertinent information, discuss relevant issues, and draw the attention of political figures. Movements’ strategic use of Twitter has increasingly been studied, though there are relatively few studies that compare social movements and their corresponding countermovements simultaneously. We examine engagement in the #DefundthePolice social movement and #DefendthePolice countermovement in a Twitter network comprised of retweets using both hashtags from August 2020 to January 2021. Text and sentiment analysis as well as a content analysis of a random sample of retweets in the network’s 20 largest subgroups reveal four key patterns. First, information commonly communicated in historical social movements is communicated in the online, Twitter network. Second, the use of movement and countermovement hashtags to criticize is common, suggesting Twitter engagement with the movement/countermovement is not a sufficient indicator of support for the movement. Third, social movements are inextricably embedded in politics, with political discourse present in all the 20 largest subgroups. Finally, though we do not include geo-tagged tweets in the analysis, physical geography is key theme in multiple subgroups. Broadly, our findings demonstrate the breadth of topics communicated within movement networks and highlight the importance of qualitatively examining Twitter data in the study of social movements.

## Introduction

In the late spring of 2020, a series of highly publicized deaths at the hands of police officers sparked national attention. The deaths of George Floyd, Breonna Taylor, Ahmaud Arbery, and Rayshard Brooks, to name the most publicized, led activists to organize large-scale protests and demonstrations in cities across the U.S. The protests publicly called for law enforcement reform of practices that were perceived to negatively impact Black communities and communities of color. More specifically, activists called on political and law enforcement leaders to critically assess and change practices associated with police officers’ excessive use of force, which they argued disproportionately resulted in the deaths of citizens of color [1].

Movements such as the Black Lives Matter [BLM] movement have been actively calling attention to racial inequalities in policing and criminal justice in the U.S. since 2013 [2]. However, the egregious nature of the highly publicized deaths in 2020 acted as a catalytic event that spurred the rapid growth of a new critical-of-police social movement, Defund the Police (defund). The defund movement presented actionable policy ideas to reduce funding for police departments and redirect those funds toward community education, healthcare, safety, and mental health resources [3]. Supporters argued this redistribution of funds would allow communities to organize and allocate resources in ways that directly benefit their citizens. As the defund movement grew, individuals who previously had no interaction or engagement with critical-of-police movements before 2020 attended protests, discussed racial injustices with friends and colleagues, and engaged with strangers on various social media sites.

The rapid expansion of and public attention given to the defund movement had an additional effect–it spurred the growth of a pro-police countermovement, Defend the Police (defend). The aim of defend was primarily to defend the actions of policing institutions and individual officers’ behaviors [4]. The defend countermovement advocated for maintaining the policing status quo, arguing that police institutions work as they are intended to and that law enforcement officers should be respected and revered for risking their lives every day to protect American citizens. Supporters viewed police agencies, police practices, and law enforcement funding as necessary to protect and preserve American democracy. In a time of heated debates over policing, the position of the defend countermovement enabled participants to react to the verbiage and claims of the defund social movement.

The social movement and countermovement literatures suggest the development and growth of the defend countermovement was a natural consequence of the proliferating defund movement [5]. The aim of a social movement is to affect broader societal change, primarily within institutions or political practices. Individuals who have less societal power must often organize to drive change through the collective action of social movements [6]. Often these efforts are aimed at acquiring members, gaining positive attention in the media, and maintaining participation in the movement until its goals are achieved [7]. Social movements target the political elite and political structures, concentrating their efforts to effect change at an institutional level [8]. Social movements become effective when they can garner mass support and cause the public to question the legitimacy and authority of leaders and the policies they support [9].

Countermovements, as is indicated in their name, rise in response to a social movements that shows signs of success [5]. At the crux of a social movement is the challenging of the status quo [10]. By default, this often leaves a part of the population which feels threatened by the social movement’s goals to change prevailing practices. If a large enough portion of the population feels threatened by a movement’s aims and has the resources to mobilize, a countermovement is formed.

Political allyship is necessary for the success of both social movements and countermovements [11,12]. Social movement scholars argue that social movements tend to be historically rooted in left-wing drives for social change (e.g., civil rights, equal pay, and abortion rights movements) [13]. Because many social movements arise to accomplish progressive goals, countermovements are historically conservative and frame their opposing movement in terms of religion, pro-family themes, and social conservatism, directing their efforts to protect the status quo and garner support from other conservatives [13]. Because social movements and countermovements both prioritize communication with political elites, the aims of both historical and modern social movements are inextricably embedded in the political context of the time [11], adding another layer of nuance to the exploration of social movements and countermovements.

Historically, recruitment to both social movements and countermovements relied largely on personal networks of members to enlist people to support and help accomplish the movement’s goals [14]. Outside of personal social networks, movements historically relied on events that produced “moral shocks” to motivate individuals to join and continue participation in the movement [15]. Jasper [16] argues that “there would be no social movements if we did not have emotional responses to developments near and far.” Emotions elicited because of moral shocks are key not only to recruitment but to driving continued participation, promoting the goals of the movement, and highlighting the importance of the efforts within social movements. The same event can cause both social movements and countermovements to engage with participants, though given the differing goals of the groups, the rhetoric and devices used to engage group members can vary notably [17]. Events that have a strong emotional impact can shift the emotional sentiment expressed within a social movement and countermovement, causing outrage or support, adding a renewed sense of commitment and value to the movement, and providing an opportunity to advocate for change directly to political elites [15,16,18].

The study of how social movements and countermovements are formed, garner support, and act to effect social change has grown to include examinations of movements on social media. The use of modern tools, such as Twitter, offer relatively new and different ways to engage with movements. Twitter is a social media site that allows users to share short (280-character) messages, called tweets. In addition to text, tweets can include photos, URL links, and Twitter handles, or mentions of other users’ Twitter usernames. Users can also retweet, like, and reply to others’ tweets. Efforts to organize meetings, protests, or other in-person opportunities are promoted by simply sharing a link to access the information to participate [19,20]. Scholars have used Twitter data to examine how individuals learn about social movements, their sentiments about said movements, and how they engage within social movement networks [7,9,21–24]. However, few studies have directly compared and investigated whether Twitter engagement and sentiment varies between social movements and their countermovements online.

The simultaneous study of an online movement and its respective countermovement poses a handful of challenges, including measuring movement support and engagement. Two recent studies separately examine the closely related social movement, Black Lives Matter, and its corresponding countermovement, All Lives Matter, and find that both supporters and opponents of Twitter-based movements engage with movements’ hashtags [23,25,26]. Gallagher and colleagues [25] find that a sizable number of tweets in an #AllLivesMatter network were written by supporters of the #BlackLivesMatter movement. These #BlackLivesMatter supporters used the countermovement hashtag to confront the views of #AllLivesMatter network members and express their disagreement. Conversely, Van Haperen et al. [26] examine this issue from the opposite perspective and find that opponents of the Black Lives Matter movement used the hashtag to identify users as targets, collectively unite, and attack their beliefs. These oppositional users coordinate their efforts to “swarm” Twitter accounts, directing their ire in an organized fashion. Thus, scholars are increasingly turning to a mix of quantitative and qualitative methods to study movements on Twitter [23,27], combating quantitative measurement issues and offering insights into the multifaceted nature of movement engagement in online forums.

It is unclear if the characteristics of engagement and sentiments within social movements and countermovements in a 21st-century, technology-dependent online environment are consistent with historical movement efforts. Twitter allows users to instantaneously communicate within and between movements and countermovements, providing an ideal tapestry to study the differences in engagement and emotional responses between social movements and countermovements during a highly politized time. This study aims to explore these topics by investigating a Twitter network of retweets using the defund and defend hashtags between August 2020 and January 2021 as a case study to examine differences in engagement and sentiment between social movements and countermovements.

## Data & methods

### Data

In this study, we examine differences in engagement and sentiment of the defund social movement and defend countermovement on Twitter. Data were collected using NodeXL, an extension of Microsoft Excel that allows users to download data using Twitter’s free API. Our data collection and analysis method complied with the terms and conditions of NodeXL. We used NodeXL Basic, a free version of the software extension, which limits the number of tweets scraped per download to either 2,000 or to all tweets from the previous eight days, capping the download at whichever parameter is reached first. We downloaded data for each hashtag separately, so for each date tweets were downloaded, the maximum number of tweets downloaded per hashtag is 2,000 or fewer if less than 2,000 tweets from the previous eight used the respective hashtag. We downloaded tweets approximately once a week over a twenty-two-week period from 8/30/20 to 1/29/21 (see Table A.1 in S1 Appendix). There were instances in both networks where the 2,000-tweet limit was not reached and NodeXL reached its download “limit” when downloads from the previous eight days were complete. When the data were merged, we removed duplicates to ensure tweets were not represented in the network twice.

Due to NodeXL Basix capping the number of tweets to 2,000 per download, the data are not comprehensive of Twitter users engaging with the defund social movement and defend countermovement. However, as we collected data approximately once a week at the same time for both hashtags, the data still offers a consistent snapshot of what was occurring in the network across both hashtags over time. Further, though the data do not include the spring/summer period in which the defend and defund movements formed and grew, the August 2020 to January 2021 period enables us to explore beyond the initial movement formation into a period that includes the 2020 Presidential election cycle. Despite these limitations, the data enable us to broadly examine patterns of engagement across and between the two movements during the 2020 Presidential election cycle.

Once all the Twitter data were collected, we combined every week of data for both hashtags into a single Twitter network representing weekly engagement with the #defundthepolice and #defendthepolice movement hashtags between August 2020 to January 2021. As discussed above, duplicate tweets were removed from the data. Our final dataset limits the tweets to retweets. The retweets include tweets that were retweeted without additional text, retweeted and had additional text added, or included additional tags of other Twitter user(s).

### Analytic strategy

We investigate the defund and defend Twitter network descriptively using multiple methodological strategies. First, we apply social network methods to investigate the network structure of Twitter engagement with the defund and defend movements between August 2020 and January 2021. Second, we apply text and sentiment analysis methods to identify the most frequently used words and the emotional sentiment expressed in tweets using the movement hashtags. Finally, we conduct a content analysis of a random subset of tweets in the largest 20 subgroups in the network to identify how actors using the hashtags engaged with the movement and countermovement. Below, we describe these three approaches in detail.

### Social network measures

First, we conceptualize the defund and defend Twitter data as a single social network in which each node represents a Twitter user engaging with one of the respective hashtags. If person A retweets person B’s tweet, there is an edge directed from person A to person B. Person A could also have an edge directed at person C if they mention person C in their retweet of person B’s tweet. We first investigate the number of nodes and edges in the network to capture how active the social movement and countermovement are on Twitter. More nodes and edges would indicate a larger number of actors engaging with the movement online.

Next, we investigate three measures of centrality: in-degree, out-degree, and degree. An actor’s indegree centrality represents the number of times they have been retweeted or mentioned in a retweet, while an actor’s outdegree centrality represents the number of times they have retweeted other actors’ tweets or retweeted other actors’ tweets while simultaneously engaging another actor in the Twitter network by mentioning them in their retweet. These measures represent the Twitter actors’ relative importance in the network and how they interact with other actors. Actors with a high in-degree centrality are popular in the network as they have a large number of Twitter users retweeting them or mentioning them in their retweets, while actors with a high out-degree centrality are highly engaged in the network as they are retweeting tweets and mentioning others in tweets as they are engaging with the hashtag. Degree centrality calculates the combined number of a node’s incoming and outgoing ties [28,29].

Finally, we examine the subgroup structure of the network visually and by measuring the modularity of the network. We use the Clauset-Newman-Moore cluster algorithm to split our nodes into subgroups. We investigate the underlying subgroup structure of the network to assess how cohesive subgroups are in the network and how interactive actors are within and between subgroups [29]. The Clauset-Newman-Moore algorithm creates subgroups using a bottom-up approach, where initially a single, individual node makes up its own subgroup. The subgroup becomes larger, containing more nodes, as that original node and the nodes it has ties to interact with more nodes in the network. This algorithm also measures the modularity score for each network which indicates how insulated subgroups are from one another. If connections between nodes within a subgroup are dense and mostly restricted to the subgroup, the modularity value is high, indicating an insular group. This measure captures how much engagement exists within and across subgroups in the networks. If the network has a high modularity score, then it would indicate that subgroups are engaging within each other using the hashtag but that the subgroups are not connecting with each other, indicating a less cohesive overall network, even if there are many users in the network.

### Text and sentiment analysis

We next conduct a descriptive text and sentiment analysis for tweets in the networks to investigate the emotional sentiments within the content being shared for the two movements. We examine the unstructured text in the tweets, and, through a tokenization process, we treat each tweet as a data frame of individual words. Thus, we treat each word in the text as a meaningful unit of text. We remove all stop words using the tidytext package in R and remove all numbers from the dataset before investigating word frequency and sentiment. We first examine the most frequently occurring words in both networks. We compare the absolute frequency of all words in the networks to examine which words occur more frequently in one network versus the other.

We then investigate the overall emotional sentiment expressed in each tweet using a sentiment analysis. A sentiment analysis systematically identifies the emotional content of language. We use an existing dataset, the Bing lexicon [30,31], created by natural language and linguistic experts, to identify whether the words used in the tweets expressed positive or negative sentiment (see Appendix for supplemental analysis). We create a measure representing the overall sentiment of each tweet by subtracting the total number of negative words in a tweet from the total number of positive words. The net value represents the overall sentiment the Twitter actor expressed in their tweet using the corresponding movements’ hashtag. Positive values represent overall positive sentiment, negative values represent overall negative sentiment, and a score of zero represents an overall neutral sentiment. We present descriptive statistics examining how the sentiment shifts during the duration of the movement in addition to identifying sentiment shifts during the weeks of three major political events: the election of Joe Biden, the Capitol insurrection, and the Presidential Inauguration (see Appendix for supplemental descriptive statistics).

### Content analysis of subgroups

We qualitatively study the network’s 20 largest subgroups to explore key topics being communicated, which hashtag is employed in the tweets, and the ideological alignment of the tweets with the social movement/countermovement. We examine a random subset of tweets using the defend or defund hashtag within each subgroup. Because of the relatively small number of tweets containing both hashtags, we do not include these in the qualitative examination of subgroups. The subgroups are created using the Clauset-Newman-Moore cluster algorithm, which identified a total of 1,716 subgroups in the combined defund and defend network, though many subgroups contained only a single node, i.e., a Twitter user retweeting themselves, or a dyadic pair.

## Results

### Network visualizations and descriptives

**Fig 1:**
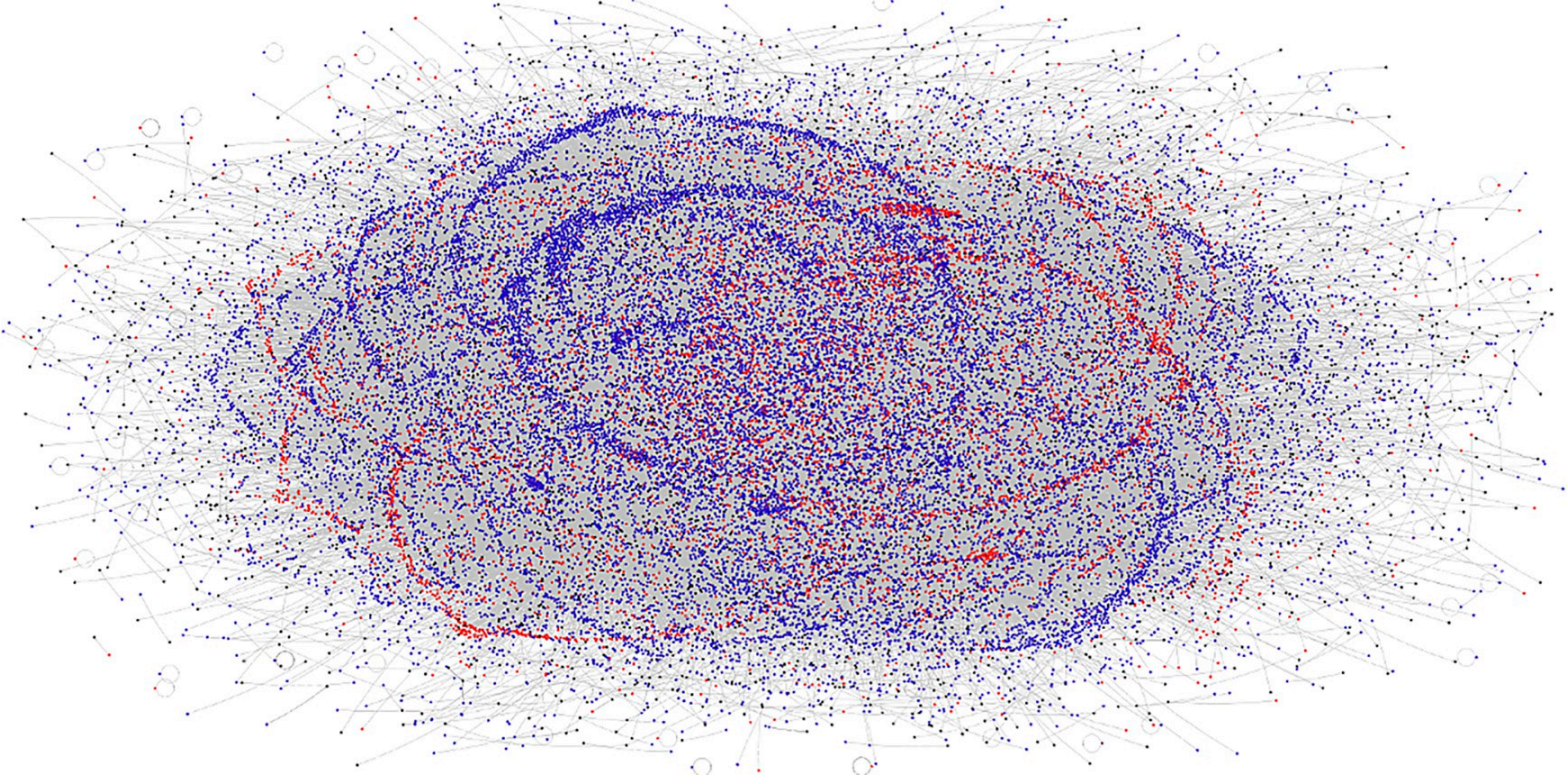
Twitter network using the hashtags #defundthepolice and #defendthepolice between 8/30/20 and 1/29/21. Note: The circles represent Twitter users in the network, and the grey lines represent retweets. The nodes are colored based on their engagement with the defund and defend hashtags. Red nodes only ever engaged with the #defendthepolice hashtag, blue nodes only engaged with #defundthepolice hashtag, and purple nodes engaged with both hashtags during the period under investigation. Black nodes are Twitter users who were only ever tagged or retweeted [have an in-degree > 0] but did not themselves engage with a hashtag in the retweet network [have an out-degree = 0]. The nodes are sized based on their out-degree centrality, with larger nodes having proportionally engaged with the hashtags more often in the network. The nodes are displayed using the force-directed Fruchterman-Reingold layout.

**Fig 2:**
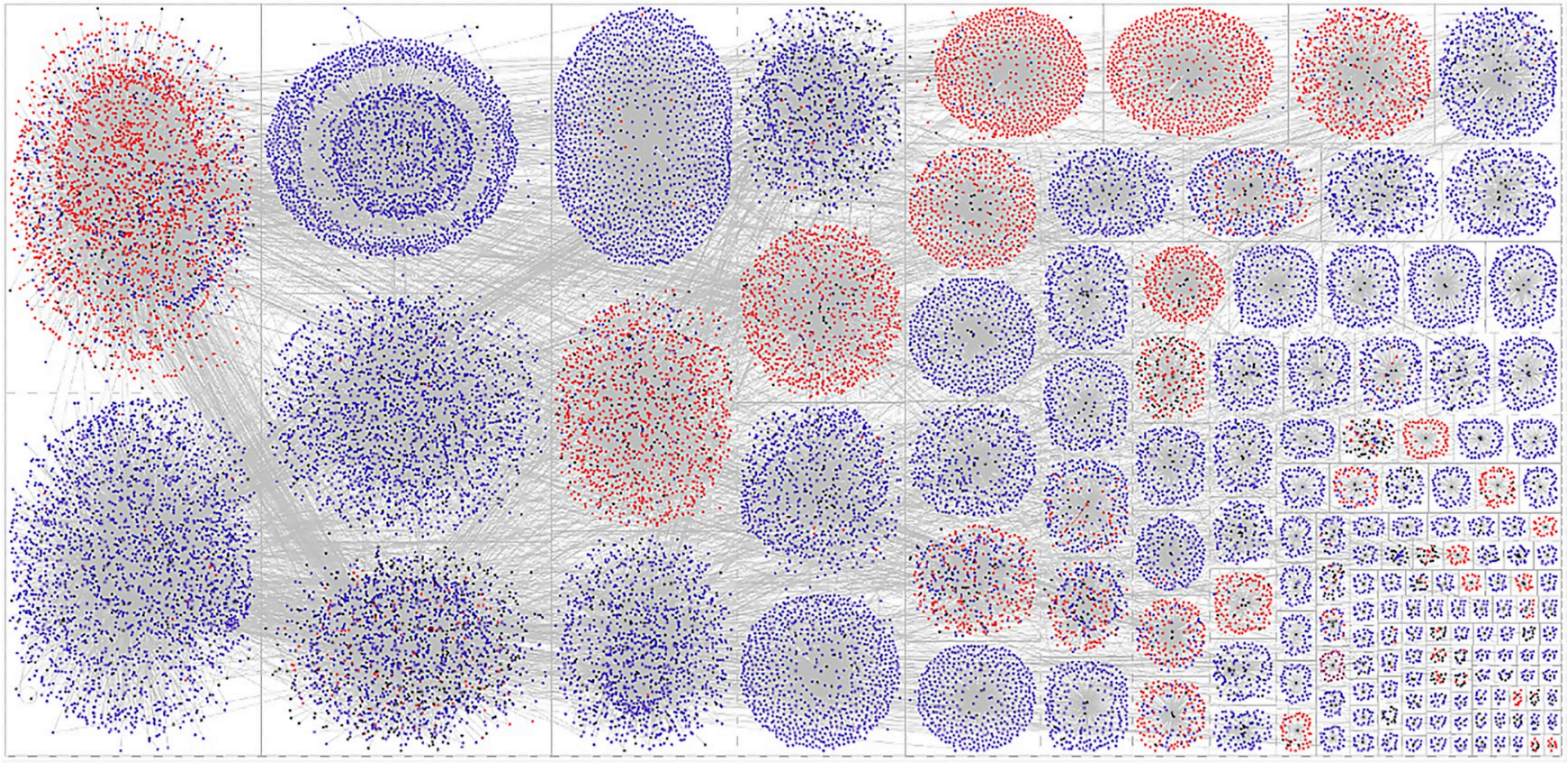
Twitter network using the hashtags #defundthepolice and #defendthepolice between 8/30/20 and 1/29/21 by subgroup. Note: The circles represent Twitter users in the network, and the grey lines represent retweets. The nodes are colored based on their engagement with the defund and defend hashtags. Red nodes only ever engaged with the #defendthepolice hashtag, blue nodes only engaged with #defundthepolice hashtag, and purple nodes engaged with both hashtags during the period under investigation. Black nodes are Twitter users who were only ever tagged or retweeted [have an in-degree > 0] but did not themselves engage with a hashtag in the retweet network [have an out-degree = 0]. The nodes are sized based on their out-degree centrality, with larger nodes having proportionally engaged with the hashtags more often in the network. The nodes are displayed using the force-directed Fruchterman-Reingold layout and partitioned into subgroups using the Clauset-Newman Moore cluster algorithm. Isolates and subgroups with 8 or fewer nodes are removed from the visualization.

Figs 1 and 2 present the network visualizations for the Twitter network of users engaging with the #defundthepolice and #defendthepolice hashtags between August 2020 and January 2021. The circles represent Twitter users in the network, and the grey lines represent retweets. The nodes are colored based on their engagement with the defund and defend hashtags. Red nodes only ever engaged with the #defendthepolice hashtag, blue nodes only engaged with #defundthepolice hashtag, and purple nodes engaged with both hashtags during the period under investigation. Black nodes are Twitter users who were only ever tagged or retweeted (have an in-degree > 0) but did not engage with a hashtag in the retweet network (have an out-degree = 0). The nodes are sized based on their out-degree centrality, with larger nodes having proportionally engaged with the hashtags more often in the network. The nodes are displayed using the force-directed Fruchterman-Reingold layout. Fig 2 presents the same network but partitions the nodes into subgroups using the Clauset-Newman Moore cluster algorithm [29]. Table 1 presents the descriptive statistics of the network statistics and overall sentiment standardized by the number of tweets engaging with the hashtags to aid in the interpretation of Figs 1 and 2.

**Table 1:** Network and sentiment descriptive statistics.

	Overall Network	Defund	Defend
Network Statistics			
Vertices	35,450	26,973	9,050
Edges	50,822	36,419	14,403
Modularity	0.85	-	-
Avg. Degree	2.65	2.57	2.75
Max Degree	2,070	2,070	10,544
Avg. In-Degree	1.33	1.29	1.38
Max In-Degree	2,062	2,062	1,050
Avg. Out-Degree	1.33	1.29	1.38
Max Out-Degree	237	237	23
Sentiment Statistics			
Avg. Sentiment	-0.08	-0.18	0.19
% Neutral Tweets	51.64%	53.25%	47.55%

Note: Sentiment is measured using the Bing lexicon.

Users engaging with the defund the police hashtag make up a far larger portion of the Twitter network during the study period than those engaging with the defend the police hashtag. Between August 30, 2020, and January 29, 2021, 26,973 Twitter users engaged with the #defundthepolice hashtag by retweeting, being retweeted, or being mentioned in a retweet, and 50,822 retweets were sent. This compares with 9,050 Twitter users and 14,403 retweets that engaged with the #defendthepolice hashtag. The defund movement was engaged with by three times as many Twitter users as the defend movement. The modularity score is very high in the network, indicating a tendency for actors to engage more within their subgroups than across the entire network. However, there are still large numbers of ties between subgroups. The users with the highest degree (2,070), in-degree (2,062), and out-degree (237) for a single actor are all found engaging with the defund hashtag. These descriptive statistics indicate that Twitter users are more engaged with the defund network than the defend network.

The subgroups analysis and the network visualization in Fig 2 shows a majority of subgroups are made up of users engaging with a single hashtag, either the defend or defund movement. However, there are several subgroups that contain both types of users indicating that there may be interaction happening between the social movement and countermovement online. We further investigate this phenomenon in the content analysis.

Table 1 indicates that half of the tweets present in the network are neutral in sentiment. The average sentiment expressed in tweets engaging with the defund network is slightly more negative than the average sentiment expressed in tweets engaging with the defend network. We explore the differences in sentiment within the networks below.

### Text and sentiment analysis

**Fig 3:**
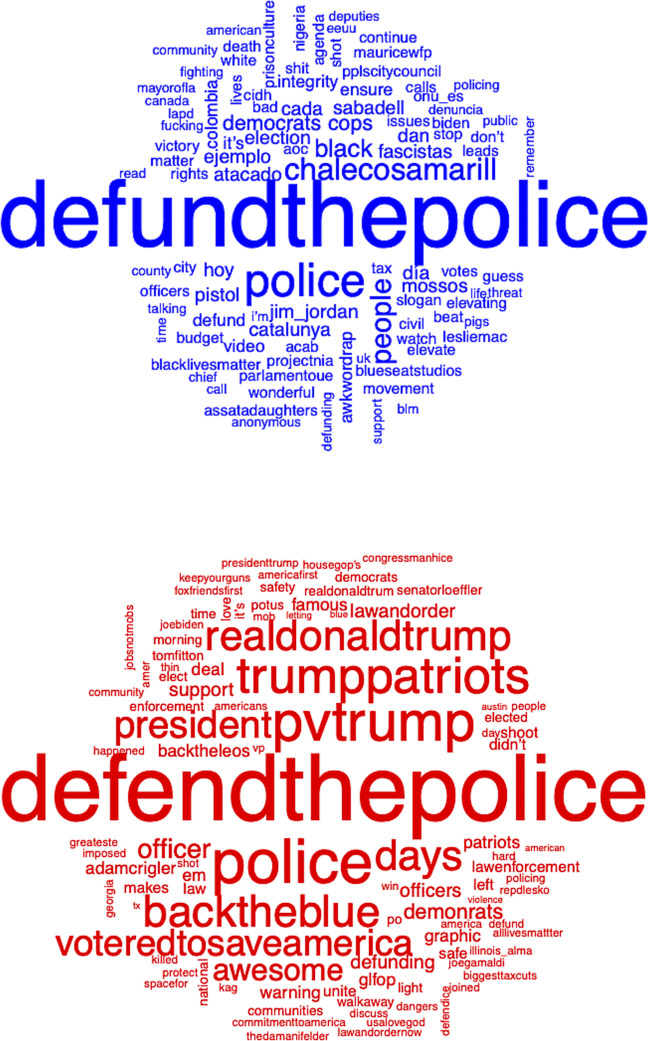
Word clouds of top 100 words in tweets using the defund and defend hashtags.

We next examine the most frequently used words and delve deeper into the patterns of sentiment across the two networks. Fig 3 presents the #defundthepolice and #defendthepolice word clouds where the top 100 most frequent words are included in the image, and the words are sized based on how frequently they occur. Though the defund network is larger and has a higher volume of actors and tweets, there is more variation in the most frequently used words in comparison with the defend network, as very few words stick out as being prominent in the network. After the hashtag itself, “police”, “people”, “black”, “democrats”, “cops”, and “election” are among the most frequent words used in the network. In contrast, the word cloud for defend indicates more consistency across the network in which words are frequently used. The words “police”, “blacktheblue”, “pvtrump”, “trumppatriots”, “voteredtosaveamerica”, “realdonaldtrump”, “days”, and “president” are prominent in the word cloud, suggesting that certain words, hashtags, and usernames are far more common in the defend network. The defend network rallies around the use of multiple hashtags, some relating to policing and others relating to politics and voting. Because of the interrelated nature of social movements, countermovements, and politics, this visually suggests that one of the countermovement’s key aims is re-electing Donald Trump for the 2020 presidency, arguably to maintain status quos in policing practices (though Donald Trump and his username, @realdonaldtrump, are frequent in the defend network, there are zero tweets from Donald Trump’s Twitter account using the hashtag #defendthepolice in the study period). Overall, the word clouds demonstrate a clear connection to politics, with references to the Republican party and its leaders being more prevalent in the defend countermovement.

**Fig 4:**
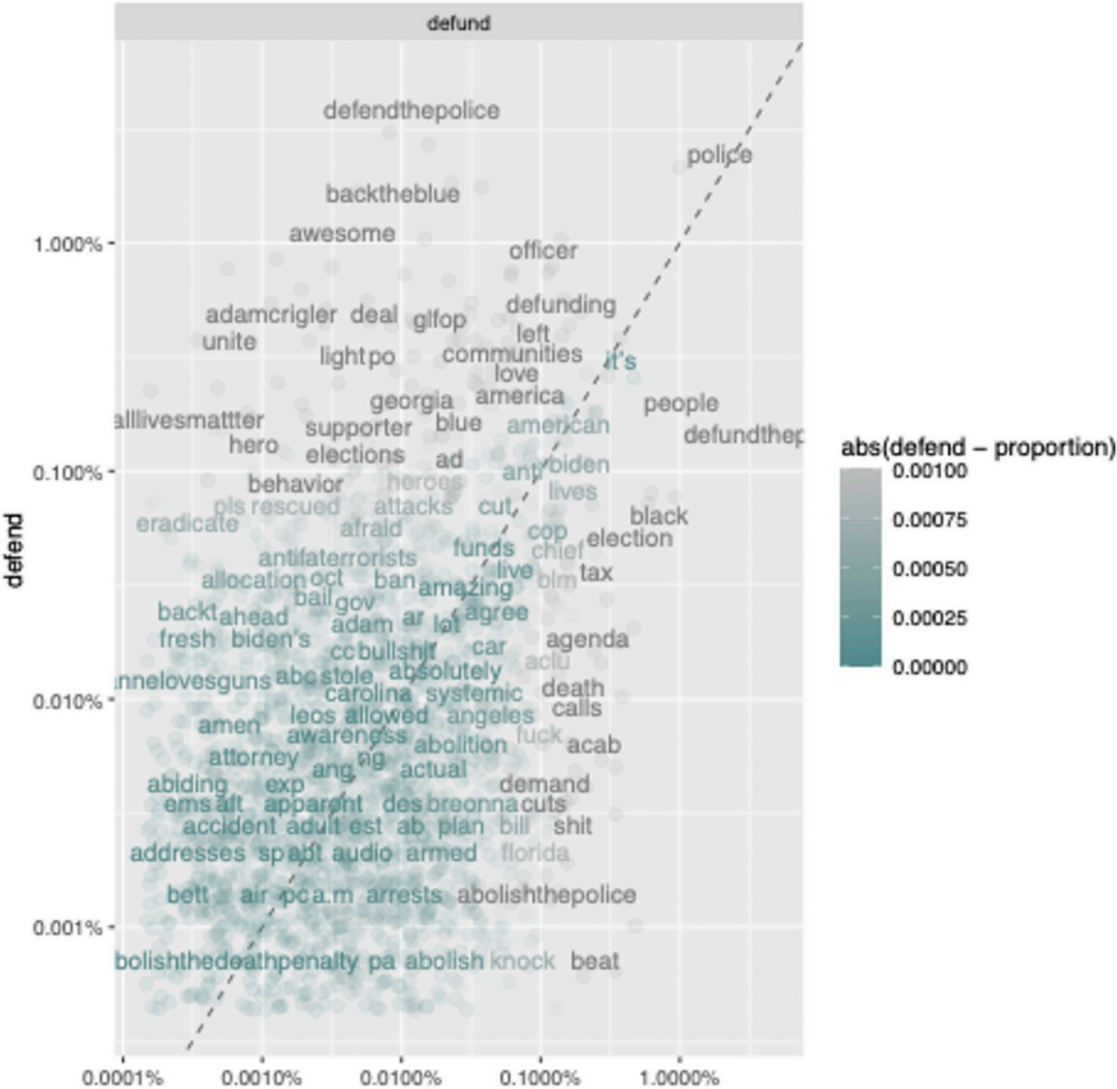
Comparison of word frequencies of tweets using the defund and defend hashtags.

In Fig 4, we present a comparison of word frequencies across the two networks. The darker gray words occur more frequently while teal words occur less frequently. Words along the diagonal occur with relatively equal frequency in both networks. Words that are above the diagonal occur more frequently in the defend network while words below the diagonal line occur more frequently in the defund network. This figure further demonstrates the politicized nature of the networks. Above the diagonal (defend) the words "supporter" and "elections" occur more, while the word "election" occurs more frequently below the diagonal (defund). Importantly, the figure also demonstrates the key messaging themes of each network. Beyond politics, users tweeting with the defend hashtag heroize the police with the words "hero", "backtheblue", and “awesome” occurring more frequently. For the defund network, the words “black”, “death”, “beat”, “tax”, “cuts”, and “abolishthepolice” occur more frequently below the diagonal, indicating the movement’s concerns that key issues need to be addressed, such as current policing tactics and budget reform.

**Fig 5:**
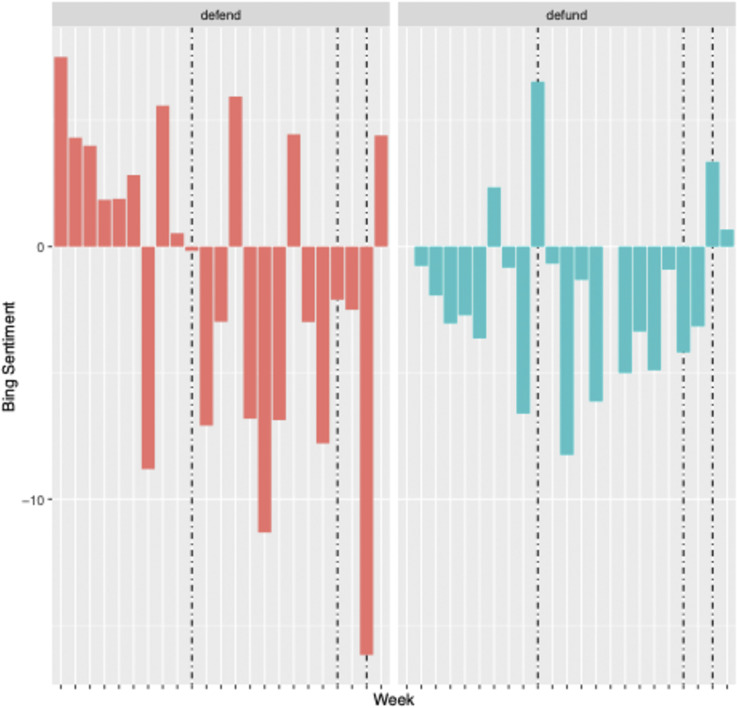
Bing sentiment of tweets using the defend and defund hashtags from 8/30/2020 to 1/29/21 with political events highlighted. Note: Political events highlighted in order are the week of the election of President Joe Biden, the week of the attack on the Capital, and the week of President Joe Biden’s inauguration.

Finally, Fig 5 presents the overall sentiment expressed in both networks over time. The y-axis represents the overall sentiment expressed in the tweets standardized by the number of tweets in the week which engaged with each hashtag, respectively, and the x-axis represents time in weeks. The dashed vertical lines indicate the three political events that, due to social movements’ political engagement, could function as emotional “moral shocks” for the movements. The first dashed line represents the week of the election of Joe Biden (11/3/20), the second dashed line represents the week of the U.S. Capitol Attack (1/6/21), and the third dashed line represents the week of President Biden’s Inauguration (1/20/21). Descriptively, this figure indicates that the defund network tends to be consistently negative across the weeks, while the defend network is more positive in August but progressively becomes more negative, with a few exceptions. Descriptively, the dashed lines suggest that these political events may have influenced the networks’ sentiment. For example, the defend network is positive in the weeks leading up to the election but becomes negative following Joe Biden’s election and is the most negative on the week of his inauguration. For the defund network each week is mostly negative until the week of Joe Biden’s election and becomes negative again until his inauguration. To better understand the topics and engagement driving sentiment in the network, we now turn to a qualitative analysis of a subset of the data.

### Subgroup content analysis

We conduct a content analysis of a random subset of retweets within the largest 20 subgroups to identify key topics discussed within the subgroups. The 20 largest subgroups include 22,092 users and 34,367 retweets. The content analysis identified four key patterns seen across the subgroups which we discuss below with their implications for social movements, countermovements, and the study of movements on Twitter.

First, most subgroups contain information that aligns with historical expectations about what is shared to promote interest in and engagement with a movement. As the network is created from tweets using #defundthepolice and #defendthepolice, all subgroups at least mention the movements via the hashtags they use. However, the network subgroups communicate varying levels of traditional movement information (TMI), such as movement updates, opportunities for involvement, and calling attention to relevant events. As Table 2 indicates, subgroups either primarily communicated TMI, somewhat communicated TMI, or simply mention TMI using the hashtags. Tweets in subgroup one, the largest subgroup, reference defund/defend only through the movement hashtags or through mentions of the defund/defend movements alongside other hot-topic political issues. In this case, the subgroup is designated as simply mentioning the movement. Of the 20 subgroups examined qualitatively, 11 were categorized as primarily movement related. Subgroups categorized as primarily movement-related are dominated by discussions of TMI.

**Table 2:** Subgroup categorizations.

	1	2	3	4	5	6	7	8	9	10	11	12	13	14	15	16	17	18	19	20
**Subgroup Movement**																				
Primarily Defund																				
Primarily Defend																				
Both																				
**Movement-Related Discourse**																				
Primarily Movement																				
Somewhat Movement																				
Mention Movement																				
**Political Discourse**																				
Primarily Political																				
Somewhat Political																				
Mention Political																				

Both the defund and defend movements shared information about their movements’ philosophies and importance. Users retweeted tweets with verbiage communicating general movement ideology, informational images, video links to interviews of movement supporters, links to informational websites, and links to protests or petitions. Other retweets simply include an emotional response to the movement, which serves as an indicator of why the user supports the movement. One such retweet states, “‘When police get to tell people what to do, they really like to tell people what to do…’ #DefundThePolice”. Though there is no information about the movement, how to get involved, or a concrete concern, a broader emotional reaction to the police is communicated, suggesting the user is uncomfortable with their perception of police officers as individuals who enjoy the act of giving orders to others.

Defund retweets sometimes include critiques of both individual police officers and general criticisms of police departments and policing. Additionally, user retweets highlight local and national budgeting decisions that could broadly impact policing. At times, calls to action were included in retweets about these issues. One retweet states, “We are into the budgeting season at @cityoftoronto. Take collective action at our Jan 25 Phone/Email Zap to demand city councilors move to defund TPS by 50%. We teach you the ropes and then work TOGETHER!”. A link is included in the retweet so users can sign up for a call/email campaign to reduce police funding in Toronto. This retweet is consistent with what we would expect from historical movements. An event, the budgeting season for Toronto, serves as a catalyst to recruit users to join movement-related efforts.

Similarly, the defend countermovement includes information about the defend philosophy and critiques of the defund movement. Defend retweets promote the heroic actions of police officers and highlight the value of police departments and policing as a system. The defend countermovement also employed fear as a tool, expressing concerns that violence would proliferate if police departments were defunded. Users retweeted “Violence is flooding our communities. Anti-police rhetoric fuels purposeful, vile attacks on #LawEnforcement officers.” This tweet highlights both general concerns of violence and concerns of violence against police officers, elucidating the importance of supporting the police. Other users retweeted a tweet stating, “Police officer has to be the most dangerous job in America these days.” While the defund movement echoes concerns about police brutality, the defend countermovement highlights the widespread risks faced by officers in the course of duty. Like the defund movement, these tweets function to elicit emotional reactions, noting the challenges faced by police officers. However, the subgroup content analysis reveals that the defend countermovement had fewer tweets that functioned purely to communicate information (see Table 2).

Second, examining the network visually, most subgroups contain retweets using both the defund and defend hashtags, though only a handful of subgroups appear to have an equal distribution of users from both hashtags. Most subgroups engage with the defund hashtag, indicating that Twitter users are engaging with the defund movement more. However, examining the content of tweets in the top 20 subgroups demonstrates that engagement does not necessarily equate support. As elaborated below, subgroup six predominately uses the defund hashtag, yet the tweets in the group overwhelmingly criticize the defund movement. When we categorize the largest 20 subgroups based on their ideological leanings, nine are categorized as primarily defund, six are categorized as primarily defend, and there are five subgroups that include multiple retweets supporting either side.

In subgroup six, many retweets employ the defund hashtag with the aim to criticize or call attention to issues within the defund movement. Though this group is engaging with defund hashtag, the content analysis suggests it is a right-wing subgroup drawing attention to concerns and issues with the defund movement. One example is a retweet of House Representative Jim Jordan’s tweet, “Democrats would rather #DefundThePolice than ensure election integrity.” Another user retweeted a right-leaning account, JudiciaryGOP, which tweeted that “Crime is coming to your neighborhood because Democrats want to #DefundThePolice.” This subgroup highlights the challenges of quantitatively studying social movements on Twitter, as the use of a movement’s hashtag in a retweet is not sufficient information to determine if a user is engaging with a network in a supportive or critical manner.

Third, while most subgroups contain movement-specific information, such as discourse about the movements’ philosophy, concerns, and organizing, subgroup one did not contain this information. This subgroup effectively demonstrates the third key finding—movements are unequivocally entrenched in politics. Political discourse occurred in all subgroups, albeit to varying degrees. As Table 2 indicates, subgroups were either primarily political, somewhat political, or simply mention politics in the course of other discussions. Of the 20 subgroups examined qualitatively, seven were categorized as primarily political. For a subgroup to be categorized as primarily political, politics dominated the subgroup to the point that there were few, if any, mentions of other movement-related topics. Subgroup one, the largest subgroup, is the clearest example of this, and many of the retweets directly referred to former President Donald J. Trump, who during the study period was a candidate for re-election. Defend users sought to re-elect Trump, tweeting, “#PresidentTrump is the #LawAndOrder @potus; @JoeBiden will never be, for the Po-po tells us so. #DefendThePolice”. Other retweets in subgroup one expressed opposing perspectives, including the defund hashtag in retweets that criticized Trump or celebrated his lack of success politically. One such tweet stated, “This is amazing! You can hear the smart people chanting ‘Vote Him Out!’ @realDonaldTrump this has to sting a lil bit!
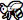
”.

As subgroup one is almost purely political in its discourse, the views expressed are predominantly about the 2020 election season occurring during the study period. Tweets in subgroup one point to an interesting pattern. Consistent with the social movement literature, movements users engage with politics to accomplish movement aims [14]. However, users also co-opt the movements’ hashtags to drive political engagement, offering limited information about the movement and instead using the hashtag to appeal to users who are assumed to be ideologically aligned with the movement or countermovement.

Subgroup five is another primarily political group, though many different politicians are mentioned in this group. Defend users in subgroup five mention specific politicians by name, such as Joe Biden, Kamala Harris, (then) Speaker Nancy Pelosi, Senator Chuck Schumer, Senator Alexandria Ocasio Cortez, Texas Governor Greg Abbott, and President Trump. Defend retweets range in their content, with users retweeting posts that tag politicians to ask how they plan to handle violence against the police and that highlight police union support for Trump. However, defend users are generally positive about right-wing politicians, supporting their campaigns more broadly and supporting right-wing verbiage on policing.

The defund users differ from defend users in this way. Among defund users, political retweets are far more likely to criticize leftist politicians or communicate concern about the 2020 Democratic candidates for President (Joe Biden) and Vice President (Kamala Harris). Users highlight concerns that Biden does not plan to take on the issue of policing while President. One retweet stated, “If you can’t get [Biden] to support #MedicareForAll or #DefundThePolice during a pandemic & 4 months of the world marching against #PoliceBrutality while on the campaign trail, you have NOTHING to push him left if he gets in office. [Biden’s] working for his 100+ billionaire owners.” Though this pattern is consistent across other subgroups, there are retweets that also laud Biden, Harris, and other leftist politicians and their aims.

Fourth, we find that geography is at times a key driver of subgroup formation, though geo-tagged tweets are not included in the analysis. Five subgroups are geographic in nature. Subgroup three is a clear example of this, as it is a highly international group focused on #defundthepolice. Retweets in this subgroup are written in multiple languages and highlight policing-related concerns globally, demonstrating the breadth and reach of the defund movement. Because of the international nature of this subgroup, there is relatively little discussion of specific politicians or political events. Instead, subgroup three primarily highlights instances of police brutality and the consequences of police brutality for a range of communities and countries. Other subgroups are specific to a country or region. Subgroups nine and 10 include both defund and defend retweets about Los Angeles, California, and Georgia/South Carolina, respectively. Retweets relating to Los Angeles broadly relate to the area, criticized police actions, politicians and/or political actions, and highlight the positive actions of law enforcement. Contrastingly, retweets in subgroup 10, relating to Georgia (GA) and South Carolina (SC), primarily criticize and praise the actions and words of two politicians, Senator Kelly Loeffler (GA) and Senator Lindsey Graham (SC).

Geography-specific subgroups four and 20 are comprised mostly of retweets about Canada, and they include information about politicians, police agency budgets, problematic police behavior, and ways to get involved with legislative decisions/politicians. The Canada subgroups are unique in that both subgroups four and 20 revolve around specific events. Subgroup four comprises almost exclusively of tweets about the budgeting process and decisions made about budgets for the Toronto Police Service, while retweets in subgroup twenty revolve around the decision in Calgary to reduce funding for the Calgary Police Service. This supports the assertion that, if concerns about or support for a movement-related event garner enough discourse, singular events can act as a catalyst to encourage movement engagement.

## Discussion

Modern social movements use social media tools, such as Twitter, to promote their messaging, acquire new recruits, and share information on movement-related topics and events. Though social movements and countermovements are widely studied on online platforms, there are few comparative studies that examine the dynamics of social movements and their corresponding countermovements alongside one another [25]. Using the #defundthepolice social movement and #defendthepolice countermovement as case studies, we examine the networks’ structure and sentiment on Twitter. Social movements historically have relied on “moral shocks” to drive recruitment and engagement efforts [15] and are inextricably embedded in the political context of the time [32]. We descriptively examine how sentiment changes over time and the most frequent words found in tweets engaging with the movements. We then qualitatively examine a random sample of tweets in each of the network’s top 20 subgroups, exploring the key topics expressed in subgroups and how users engaged with each hashtag.

In preliminary examinations of word frequencies and sentiment across the two networks, the differing dynamics between the defund and defend networks are stark. The defund network has more actors and engagement, is more negative in sentiment, and appears to be highly aware of both highlighting concerns regarding policing and noting the political nature of the problem. Words such as “police”, “people”, “black”, and “watch” are common, indicating the focus on issues of policing and problems faced disproportionately by communities of color. Though less prominent, the words “election” and “democrats” occur with relatively high frequency in the defund network, indicating the movement’s awareness of and engagement with the political environment of the time. Further, the sentiment of the defund network is relatively negative and consistent over time. This could indicate the continued focus on the goal of the movement, reducing police violence and power across communities.

The defend network is smaller, has greater shifts in sentiment over time, and is arguably more political in nature. Though defend actors actively discuss law enforcement practices and police officers, as evidenced by the frequency of the words “police”, “backtheblue”, and “officer”, political words dominate the network. Frequent words in defend include specific mentions of voting and Donald Trump, such as “realdonaldtrump”, “pvtrump”, “president”, “trumppatriots”, and “voteredtosaveamerica”. Additionally, the sentiments expressed in defend network tweets decline significantly over time. Over the course of the 2020 election season and the 2021 inauguration of Joe Biden, sentiments in the defend network transition from largely positive to largely negative.

The findings for the descriptive word frequencies and overall sentiment shifts are largely consistent with expectations from the social movement literature. The defund network appears to be primarily focused on its messaging, highlighting problematic police practices, the relative disadvantages Black Americans face, and pushing for changes to law enforcement institutions. Defund also situates itself in the political landscape, discussing the 2020 election and the Democratic party, the largest left-wing party in the U.S. Both messaging and tone appear to stay relatively constant, as evidenced by the consistent, negative sentiment throughout the period. The goal of social movements is to drive social change [10,15] and the characteristics of the defund network suggest that the goals of the movement remain at the forefront of the discussion over time.

Alternatively, the defend network discusses the police, but political verbiage and words were far more prevalent. Additionally, the sentiment in the network decreased drastically over the period. It appears that, because the defend network’s aim is to maintain the status quo law enforcement practices, messaging in the network is focused on political efforts to re-elect Donald Trump. It is possible that, for actors in the defend network, the election of Donald Trump represented the best chance defend had at limiting changes to policing practices. This is consistent with the expectations of a countermovement [7]. Further, the decline in sentiment over time corresponds with shifts in the 2020 election cycle. The 2020 presidential election season between the Democratic (Joe Biden) and Republican (Donald Trump) indicated a close race, and many Trump supporters were enthusiastic about his chances of re-election. However, when his re-election did not come to fruition, enthusiasm in the defend network dimmed.

The subgroup content analysis offers insight into the communications within the defund and defend movements. Information that is historically associated with social movement communications was conveyed within the network, indicating that the capabilities of Twitter are harnessed to facilitate quick, widespread movement information. This includes emotional reactions to the movement, which are historically invoked to garner support for the movement. However, the use of opposing hashtags was common, suggesting users are employing these hashtags to promote discussion rather than to promote their individual allegiance to a movement. This finding is consistent with past work [23,25,26], suggesting that using a movement’s hashtag is not sufficient evidence of support, a reality that must be considered in future Twitter studies of social movements.

Though many retweets within the network include messaging about the goals of the movement, how to get involved, and the importance of the movement, politics reigned supreme as the key theme in all subgroups. No subgroup was untouched by politics. Both the movement and countermovement demonstrate the importance of political engagement for movements through the frequent discussion of political decisions, mention of political parties, and tagging of specific politicians. Further, the content analysis suggests the interaction of movement tone and political discourse could be driving differences in sentiment between defund and defend movements. Finally, though we did not have geographic identifiers for the retweets in the analysis, subgroup formation occurred around geographic areas when groups of users retweeted international or location-specific movement developments. This suggests that movement networks on social media can both elevate the importance of local issues and demonstrate the movement’s importance on a global scale.

There are three implications regarding the differences in political messaging and the tone, language, and messaging of retweets using the defend and defund hashtags. First, this is largely consistent with the expectations of historical social movements. The defund movement aims to call attention to issues relating to policing and to the lack of political action taken to address these issues. Political critiques offer an avenue for defund to accomplish their movement aims, highlighting past and present issues to drive future change. The defend countermovement, however, aims to maintain the status quo, and positive messaging and reference to right-wing political groups or politicians can help to accomplish this.

Second, studies of social movements and countermovements suggest that movements use their collective efforts to draw support from politicians [14]. Communication with politicians is then used as a tool to share the movements’ aims and appeal to those in positions of power who could forward these aims through impactful policies and public efforts. However, findings from the qualitative analysis suggest that users also co-opt the movements’ hashtags to gain support for political figures. This is especially true in the largest subgroup, subgroup one, as the tweets in subgroup one are primarily aimed at communicating election-related information and include minimal substantive information about the defund and defend movement goals and efforts.

Third, the differences in tone and the ubiquitous nature of political messaging in the network can offer insight into differences in sentiment between hashtags. Defend retweets are overall more positive, yet sentiment declines steadily in the study period. The subgroup content analysis suggests that defend political messaging prior to the 2020 election is highly positive, denoting excitement for the upcoming election and confidence that Trump would be re-elected. Because of the volume of political tweets in the defend hashtags, this positive messaging likely inflates sentiment scores for the defend hashtag. However, defund users are critical to politicians through two avenues. Supporters of the defund movement are far more likely to criticize left-wing politicians than defend supporters are to criticize right-wing politicians, *and* defund critics who use the defund hashtag offered additional criticisms of left-wing politicians and politics. Both the negative political messaging and the overall critical nature of the movement contribute to the steady, slightly negative sentiments expressed in the defund tweets.

These findings should be viewed in the context of some important study limitations. One key limitation of the analysis is the data are not comprehensive across the study period for the combined #defundthepolice and #defendthepolice network. Due to funding limitations for this project, we opted to use NodeXL Basic to download the data for the analysis, and this limited the number of tweets downloaded at a time to 2,000. Thus, the data represent a snapshot of engagement across the defund movement and the corresponding defend countermovement. Most critically, the entire universe of retweets is not included in the data, potentially biasing estimates of sentiment within the network. However, the qualitative examination of the 20 largest subgroups did not reveal any significant communication gaps, suggesting that constructing a network of retweets allowed us to capture adequate variation over time in the network.

Despite this limitation, there are advantages to using NodeXL. If we retroactively collected the data, tweets that were deleted after their initial posting would not be included, so the data have the advantage of including tweets that have been subsequently deleted or were posted from accounts that were removed from Twitter altogether. Additionally, across the study period, we aimed to collect data approximately once a week, offering consistent snapshots of what is occurring in the network over time (see Table A.1 in S1 Appendix). The data were downloaded manually, and we downloaded data on Fridays for 16 of the 21 downloads to capture data at similar times each week.

We did not collect data in the late spring and summer of 2020, so we could not examine the growth of the network or sentiment changes since its inception. Moreover, the descriptive nature of our analysis precludes us from making causal claims on the relationship between politics and movements. Our study only examines the defund and defend network on Twitter which may not reflect the movements’ engagement and sentiment shared in-person and on other platforms. Despite the limitations, the data enable us to broadly examine patterns of engagement across and between the two movements, examine the substantive topics discussed in the network’s subgroups, and explore how and why movements invoke and interact with politics while promoting their aims.

We encourage further research that explores online networks for social movements and countermovements. We did not examine shifts in the structure of the networks over time. The social movements literature suggests that membership and engagement within networks is fluid, and it is possible examining changes in network structure over time would better allow researchers to understand social movements, countermovements, and the differences between them. Given the international nature of the Defund the Police movement and the geography-specific subgroups, we suggest both quantitatively and qualitatively examining content from online platforms, such as Twitter, to better understand differences across places in engagement with social movements and countermovements. We qualitatively examine the top 20 subgroups as a descriptive step in this direction, though delving deeper into the network data would enable a better understanding of the language choices of actors, patterns of engagement across the networks, and key topics discussed within movements across places.

## Supporting information

S1 Appendix Table A.1 Dates of Data Downloads for #DefundthePolice and #DefendthePolice Using NodeXL Basic. (DOCX)

S1 Fig Figure A.1. Top 50 Most Frequent Words in Tweets using the Defend and Defund Hashtags.
(TIFF)

S2 Fig Figure A.2. Distribution of Bing Sentiment of Tweets using the Defend and Defund Hashtags.
(TIFF)

S3 Fig Figure A.3. Distribution of AFINN Sentiment of Tweets using the Defend and Defund Hashtags.
(TIFF)

S4 Fig Figure A.4. AFINN Sentiment of Tweets using the Defend and Defund Hashtags from 8/30/2020 to 1/29/21 with Political Events Highlighted.
(TIFF)

S5 Fig Figure A.5. Bing Sentiment of Tweets using the Defend and Defund Hashtags from 8/30/2020 to 1/29/21 with Police Excessive Use of Force Events Highlighted.
(TIFF)

S6 Fig Figure A.6. AFINN Sentiment of Tweets using the Defend and Defund Hashtags from 8/30/2020 to 1/29/21 with Police Excessive Use of Force Events Highlighted.
(TIFF)

S1 Data (CSV)
