# Supplementary material for: From an election to an insurrection: Investigating differential engagement and sentiment in the #defundthepolice and #defendthepolice network on Twitter
Source: PLoS One. 2024 Mar 21;19(3):e0289041. doi: 10.1371/journal.pone.0289041 (PMC10956855; doi:10.1371/journal.pone.0289041)
Supplement: S1 Appendix — (DOCX) [file pone.0289041.s001.docx]

**Appendix**

| **Table A.1 Dates of Data Downloads for #DefundthePolice and #DefendthePolice Using NodeXL Basic** | |
| --- | --- |
| **Date** | **Day of the Week** |
| 8/30/20 | Sunday |
| 9/6/20 | Sunday |
| 9/11/20 | Friday |
| 9/25/20 | Friday |
| 9/30/20 | Wednesday |
| 10/4/20 | Sunday |
| 10/9/20 | Friday |
| 10/23/20 | Friday |
| 10/30/20 | Friday |
| 11/6/20 | Friday |
| 11/13/20 | Friday |
| 11/20/20 | Friday |
| 11/27/20 | Friday |
| 12/4/20 | Friday |
| 12/19/20 | Saturday |
| 12/25/20 | Friday |
| 1/1/21 | Friday |
| 1/8/21 | Friday |
| 1/15/21 | Friday |
| 1/22/21 | Friday |
| 1/29/21 | Friday |

Figure A.1. Top 50 Most Frequent Words in Tweets using the Defend and Defund Hashtags.

Figure A.2. Distribution of Bing Sentiment of Tweets using the Defend and Defund Hashtags.

Figure A.3. Distribution of AFINN Sentiment of Tweets using the Defend and Defund Hashtags.

Figure A.4. AFINN Sentiment of Tweets using the Defend and Defund Hashtags from 8/30/2020 to 1/29/21 with Political Events Highlighted.

Note: Political events highlighted in order are the week of the election of President Joe Biden, the week of the attack on the Capital, and the week of President Joe Biden’s inauguration.

Figure A.5. Bing Sentiment of Tweets using the Defend and Defund Hashtags from 8/30/2020 to 1/29/21 with Police Excessive Use of Force Events Highlighted.

Note: Police excessive use of force events highlighted in order are the week of the Kentucky Grand Jury Indictment of 1 of 3 Officers in Breonna Taylor’s Case on September 23, 2020; the week of the Seattle Office of Police Accountability’s publication of findings that Seattle police officers used excessive force with protestors on October 23, 2020; and the week of the shooting of Walter Wallace Jr. on October 26, 2020.

Figure A.6. AFINN Sentiment of Tweets using the Defend and Defund Hashtags from 8/30/2020 to 1/29/21 with Police Excessive Use of Force Events Highlighted.

Note: Police excessive use of force events highlighted in order are the week of the Kentucky Grand Jury Indictment of 1 of 3 Officers in Breonna Taylor’s Case on September 23, 2020; the week of the Seattle Office of Police Accountability’s publication of findings that Seattle police officers used excessive force with protestors on October 23, 2020; and the week of the shooting of Walter Wallace Jr. on October 26, 2020.
