# Supplementary figures and images for: From an election to an insurrection: Investigating differential engagement and sentiment in the #defundthepolice and #defendthepolice network on Twitter
Source: PLoS One. 2024 Mar 21;19(3):e0289041. doi: 10.1371/journal.pone.0289041 (PMC10956855; doi:10.1371/journal.pone.0289041)

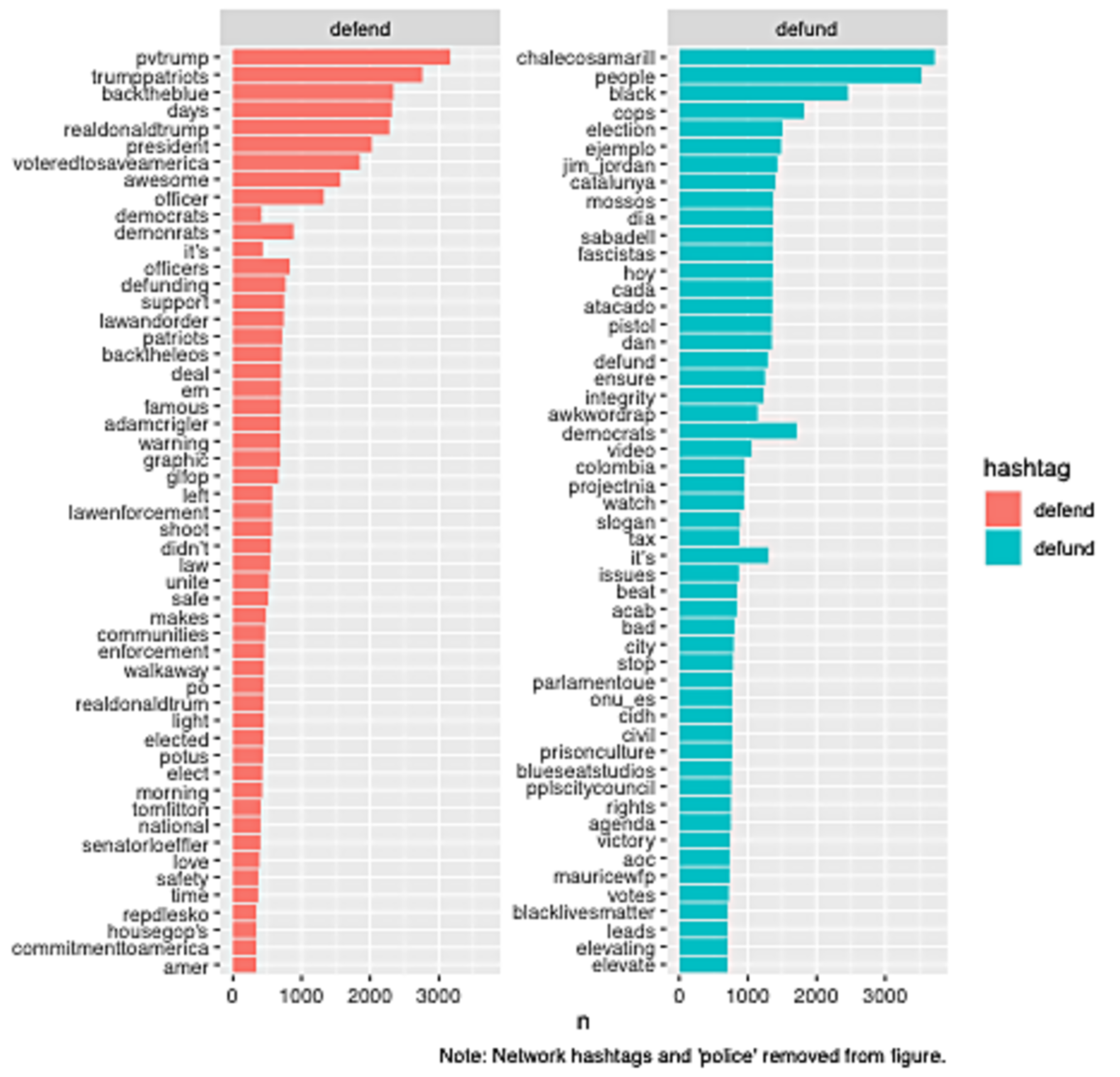

Supplement: S1 Fig — Top 50 Most Frequent Words in Tweets using the Defend and Defund Hashtags. (TIFF) [file pone.0289041.s002.tiff]

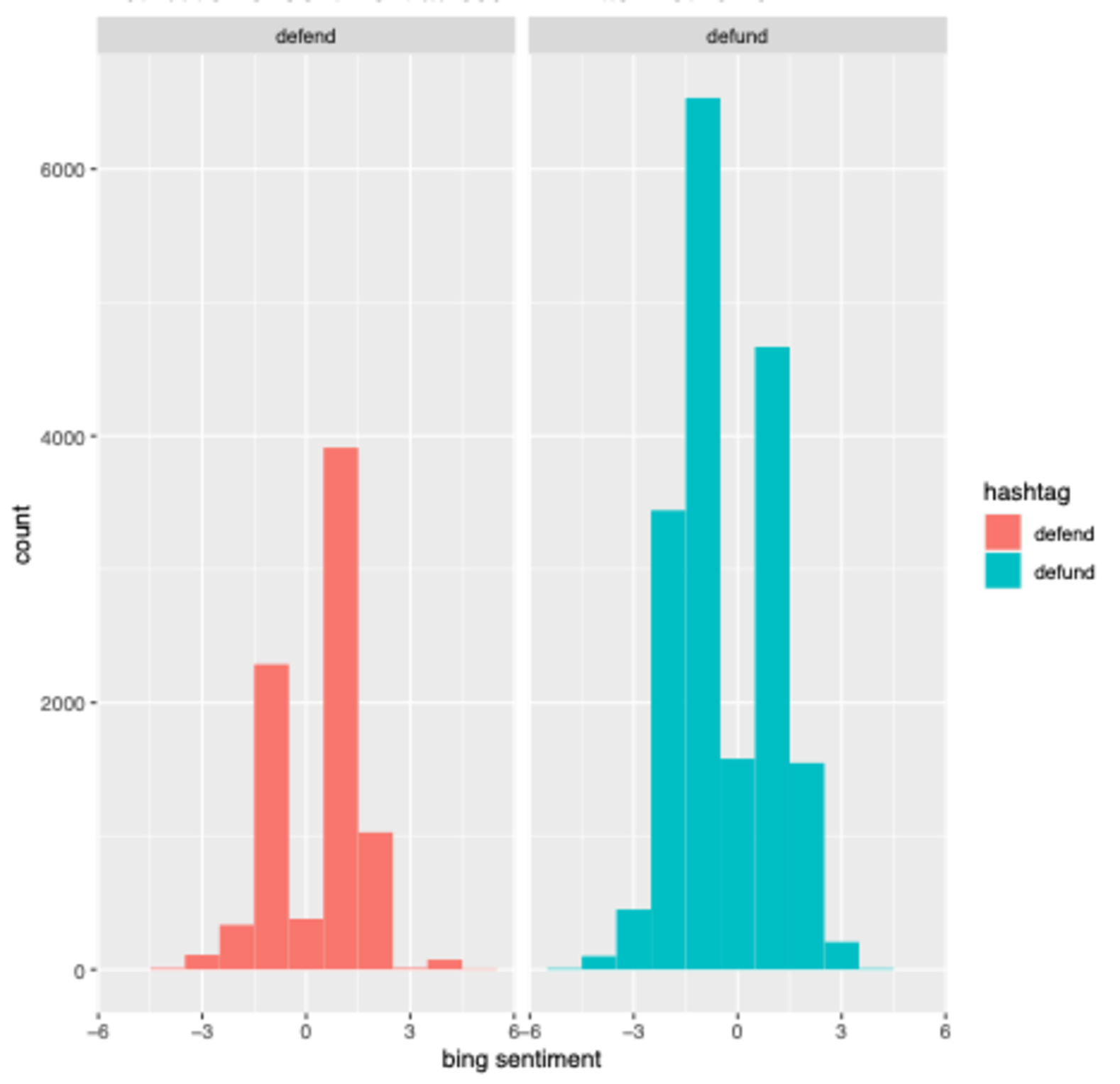

Supplement: S2 Fig — Distribution of Bing Sentiment of Tweets using the Defend and Defund Hashtags. (TIFF) [file pone.0289041.s003.tiff]

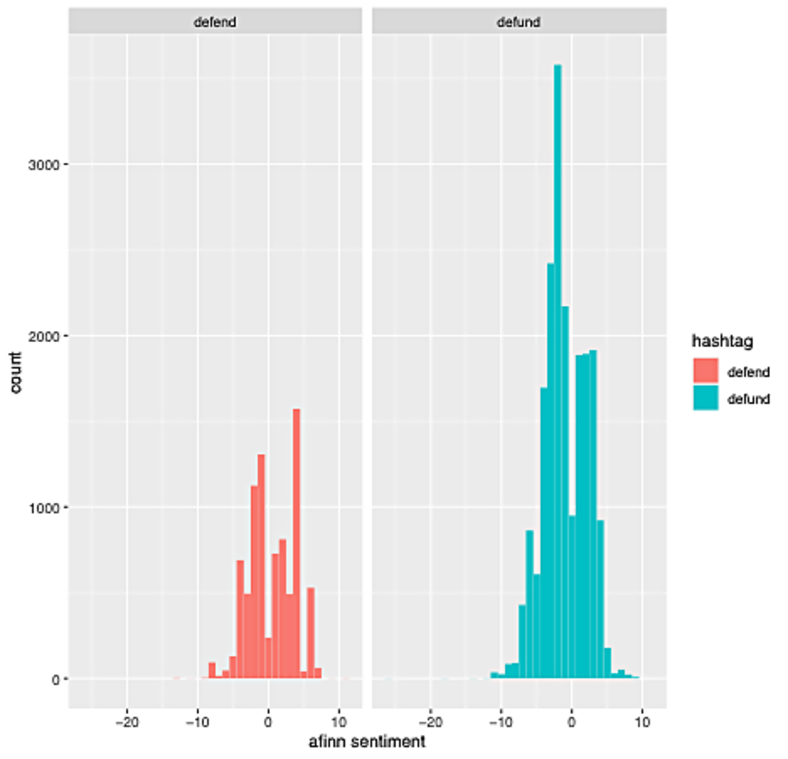

Supplement: S3 Fig — Distribution of AFINN Sentiment of Tweets using the Defend and Defund Hashtags. (TIFF) [file pone.0289041.s004.tiff]

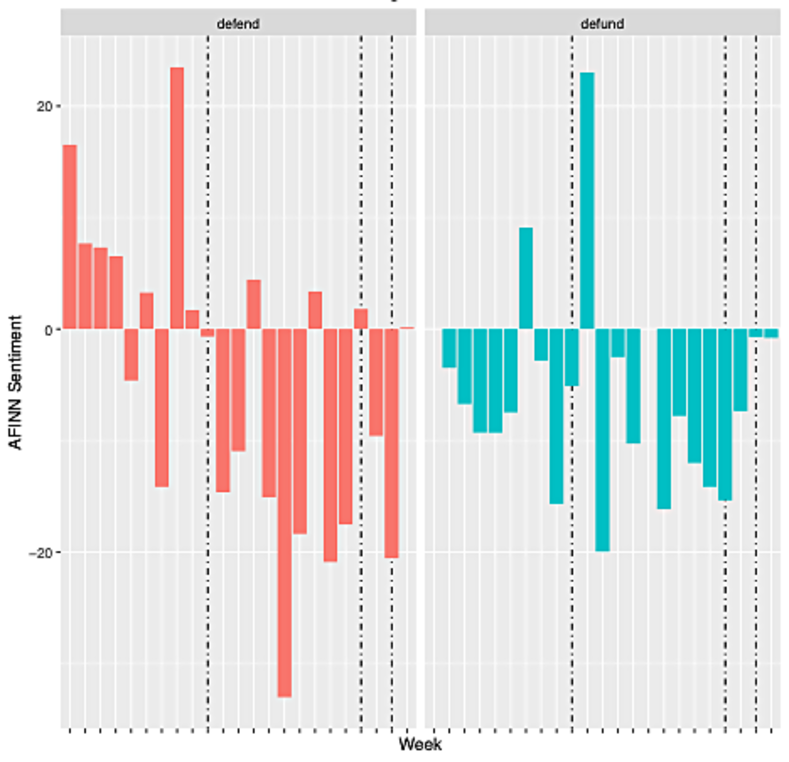

Supplement: S4 Fig — AFINN Sentiment of Tweets using the Defend and Defund Hashtags from 8/30/2020 to 1/29/21 with Political Events Highlighted. (TIFF) [file pone.0289041.s005.tiff]

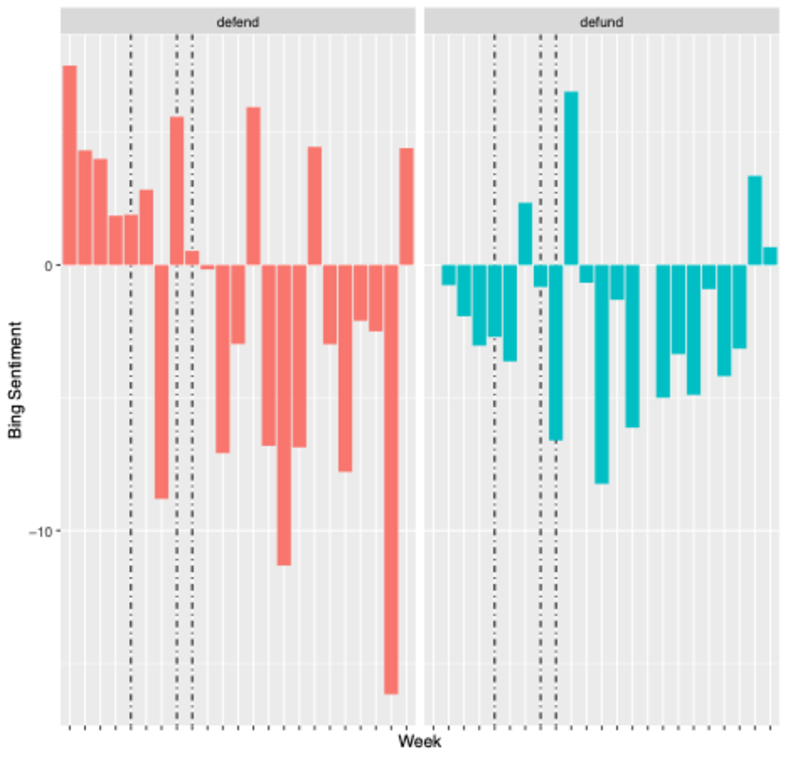

Supplement: S5 Fig — Bing Sentiment of Tweets using the Defend and Defund Hashtags from 8/30/2020 to 1/29/21 with Police Excessive Use of Force Events Highlighted. (TIFF) [file pone.0289041.s006.tiff]

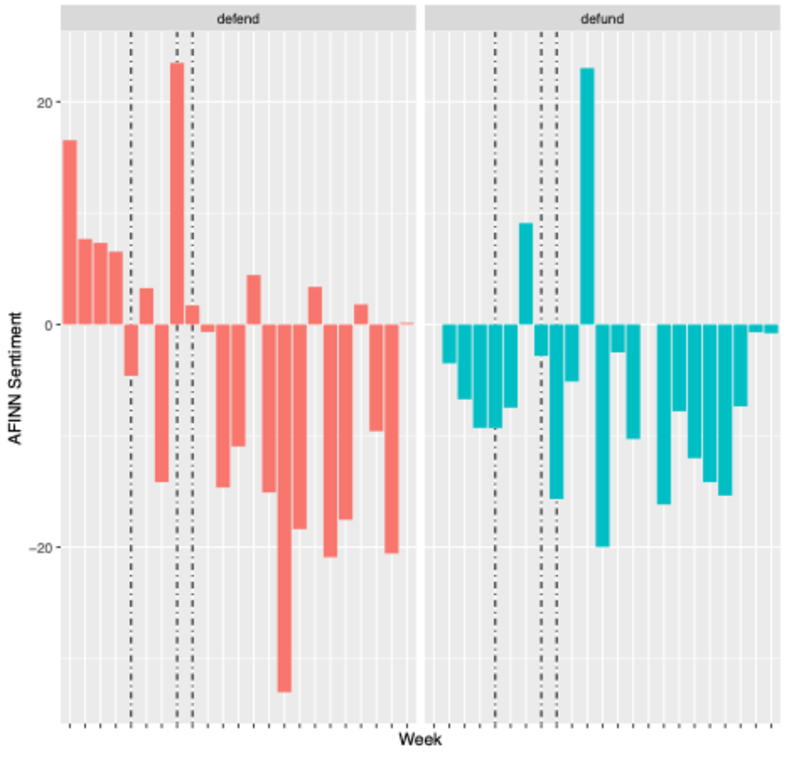

Supplement: S6 Fig — AFINN Sentiment of Tweets using the Defend and Defund Hashtags from 8/30/2020 to 1/29/21 with Police Excessive Use of Force Events Highlighted. (TIFF) [file pone.0289041.s007.tiff]
